# GPU-Q-J, a fast method for calculating root mean square deviation (RMSD) after optimal superposition

**DOI:** 10.1186/1756-0500-4-97

**Published:** 2011-04-01

**Authors:** Ling-Hong Hung, Michal Guerquin, Ram Samudrala

**Affiliations:** 1 Department of Microbiology, University of Washington, Seattle WA USA

## Abstract

**Background:**

Calculation of the root mean square deviation (RMSD) between the atomic coordinates of two optimally superposed structures is a basic component of structural comparison techniques. We describe a quaternion based method, GPU-Q-J, that is stable with single precision calculations and suitable for graphics processor units (GPUs). The application was implemented on an ATI 4770 graphics card in C/C++ and Brook+ in Linux where it was 260 to 760 times faster than existing unoptimized CPU methods. Source code is available from the Compbio website http://software.compbio.washington.edu/misc/downloads/st_gpu_fit/ or from the author LHH.

**Findings:**

The Nutritious Rice for the World Project (NRW) on World Community Grid predicted *de novo*, the structures of over 62,000 small proteins and protein domains returning a total of 10 billion candidate structures. Clustering ensembles of structures on this scale requires calculation of large similarity matrices consisting of RMSDs between each pair of structures in the set. As a real-world test, we calculated the matrices for 6 different ensembles from NRW. The GPU method was 260 times faster that the fastest existing CPU based method and over 500 times faster than the method that had been previously used.

**Conclusions:**

GPU-Q-J is a significant advance over previous CPU methods. It relieves a major bottleneck in the clustering of large numbers of structures for NRW. It also has applications in structure comparison methods that involve multiple superposition and RMSD determination steps, particularly when such methods are applied on a proteome and genome wide scale.

## Background

### Structure comparison by optimal superposition and RMSD calculation

In order to compare two protein structures, a transformation is first obtained that optimally superposes corresponding atoms from the one structure onto the other. The root-mean-square- deviation (RMSD) between the coordinates of the superposed structures is then calculated [1]. In structural biology, such comparisons are very common and RMSD is used as shorthand for root mean square deviation after optimal superposition. RMSD calculations form the basic building block for more sophisticated structural comparison methods that also optimize the subset of atoms being compared. This is desirable when there may be divergent regions that would dominate the superposition. Multiple RMSD calculations using different possible sequence mappings are also necessary when a new protein is being compared to a library of known folds to help determine its function [2-4].

For genome wide applications, such as the Nutritious Rice for the World (NRW) project, structure comparison becomes a bottleneck for the clustering of large ensembles of candidate structures on the basis of structural similarity. Consumer graphic processing units (GPUs), designed to rapidly process video for games and home entertainment, utilize many individual processors that operate simultaneously. This results in TFLOPS (10^12 ^floating operations per second) speeds which are 2 to 3 orders of magnitude faster than consumer CPUs. GPUs are becoming ubiquitous and their impact is being felt especially in community grid projects which utilize home machines. To access this power, methods must be specifically adapted to GPUs, to take into account their characteristics, such as the need for independent threads, their optimizations for single precision vector operations, and penalties for branching and memory fetches of data.

The simplest method for optimal superposition translates the center of masses or barycenters of the two molecules to the origin and then applies a rotation to one molecule that minimizes the covariance matrix between the two sets of coordinates [1]. An equivalent formulation uses quaternions and reduces to solving for the eigenvalues and eigenvectors of a 4 × 4 matrix [5]. A key consideration in the choice of a quaternion-based methodology was that the rotational method (Rot) for RMSD calculations requires at least two fetches of coordinates whereas the quaternion method can be implemented with just one. All quaternion based methods solve for the eigenvalues of a symmetric 4 × 4 matrix derived from the covariances between the coordinates of the two sets of atoms being compared. The maximum eigenvalue is used to obtain the RMSD. The fastest of previously published methods, Q-CP, does this by finding the largest real root of the characteristic polynomial associated with the matrix, using a Newton-Raphson solver[6,7]. Unfortunately, all the variants that were tried in formulating and solving the quartic characteristic polynomial proved to be unstable with single precision calculations.

Coutsias and co-workers [5] obtained the eigenvalues and eigenvectors of the 4 × 4 matrix using the Power algorithm (Q-P). However, this was also the slowest CPU implementation that we tested even though it was implemented in FORTRAN. Instead, we used the cyclic-Jacobi method [8] for solving for the eigenvalues in our implementations of the quaternion-based algorithm (Q-J and GPU-Q-J). Q-J is almost as fast as Q-CP on CPUs, and converges stably even with single precision calculations.

### The test set - Nutritious Rice for the World on World Community Grid

Current implementations are very fast and more than sufficient for routine applications where only a few RMSD calculations are necessary. However, for applications involving very large datasets, current RMSD methods become a significant bottleneck. Our implementation was motivated by the needs of one such project, the Nutritious Rice of the World, (NRW) [9]. NRW ran on World Community Grid [10], a distributed computational project that utilizes the resources of a community of volunteers. It was created by IBM in 2004 and by the end of 2009, there were almost 500,000 members registered over 1.5 machines on the grid providing over 300,000 years of computing time in total. World Community Grid has supported many different projects, one of them being NRW. Users download our ProtinfoAB client program [11,12], which requests workunits from World Community Grid servers, processes them using idle computing cycles, and returns the structure predictions to World Community Grid. The aim of NRW was to predict the structure of all small rice proteins from their sequence. The predicted structures can then be screened against known protein fold families to identify the function of the gene product. 25,761 CPU-years were devoted to computing 10 billion structures for NRW. These results would have taken our local cluster over 30 years to obtain but took less than 2 years using World Community Grid.

Our methodology for choosing the best candidate structures from the 62,000 ensembles involves clustering the candidate structures using RMSD as a metric [13,14]. The construction of the similarity matrix consisting of the RMSD between every pair of structures is an O(N^2^) process. For a typical set of 100,000 structures generated by NRW for a single sequence, this would take days on a CPU. We filter the ensemble using different energy functions [4,5] to reduce the size to the 5000 best structures before clustering. To cluster even this smaller set can take an hour on a CPU. As shown in Additional file 1: Supplemental Table S1, this could be accomplished in a few seconds using a GPU. There are different sets of energy functions that can be used to filter the proteins to produce different subsets. In addition, it would be preferable to cluster larger numbers of structures. The CPU implementations of RMSD calculations, while more than fast enough for most applications, become a bottleneck when examining 62,000 ensembles of structures.

## Results and Discussion

### Algorithm

The quaternion algorithm is described in detail in [5] so we will only review it briefly. Let the position vectors of the k_th _atoms for the two proteins be:

The covariance matrix for a comparison of *N *atoms is given by

The terms can be calculated with one fetch of coordinates by re-arranging to:

A 4 × 4 symmetric matrix can be obtained:

The RMSD after optimal superposition is then given by:

where *λ_max _*is the maximum eigenvalue of the 4 × 4 matrix.

The eigenvalues were obtained using a modified version of the standard library implementation of cyclic-Jacobi method[15]. GPU subroutines are actually inline substitution of code and not implemented through a stack. Thus the same processor constructs the matrix and calculates the eigenvalues. It is not possible to parallelize both processes independently. Moreover, there is only limited memory for code and variables. Branching in GPUs can also be very slow. Therefore we coded the cyclic-Jacobi method specifically for a symmetric 4 × 4 matrix and minimized the number of variables, instructions and branch points.

### Implementation

#### Hardware details

The GPU-Q-J algorithm was implemented on an ATI 4770 graphics card. The ATI 4770 GPU contains 640 stream processors with access to 512 MB of onboard GDDR5 memory. The processors are organized into groups of 5 consisting of 4 standard stream processors and one with specialized trigonometric capabilities. Single precision floating operations use all stream processors simultaneously and double precision operations use a group of 5 to generate a single result. Thus, double precision operations are at least 5 times slower. The card is a rated at approximately 1 TFLOPS for single precision calculations, 200 GFLOPS (10^9 ^floating operations per second) for double precision calculations and consumes 80W at peak. Communication between the CPU and GPU was through a PCIe 16 bus. A single core Sempron 3000+ system (running Linux), was used in test applications so that thread management among multiple cores would not factor into the comparison. The CPU scored 1.3 GFLOPS in double precision multiplication and addition.using flops.c [16] as a benchmark.

#### Software environment

The ATI SDK 1.4 provides Brook+ [17] as a high level language to interface with its GPUs. Brook+ is based upon Brook [18], a C/C++ like language developed at Stanford. The main extension to standard C/C++ is the stream data structure. Stream data is organized and indexed in a manner similar to regular arrays which are the mechanism for supplying inputs and capturing outputs. In a GPU accelerated process, the CPU writes data into input streams to be sent to the graphics card. An onboard job scheduler assigns an index to a set of stream processors. The data in the input streams at that index are read in, operated on and then output to one or more output streams with the matching index. The numerous stream processors can operate simultaneously on their assigned pieces of the data streams which results in the acceleration.

More recent ATI GPUs support "gather" and "scatter" streams. A gather stream is a data structure where stream processors can read or gather data from any piece of the stream. A scatter stream is the output analogue where the stream processors can write or scatter data to any piece of the stream. Combined scatter/gather streams are also available which allow random access reads and writes from and to the same stream data structure. Gather and especially scatter streams are slower than regular streams but are much more flexible.

#### GPU optimizations

**Figure 1:**
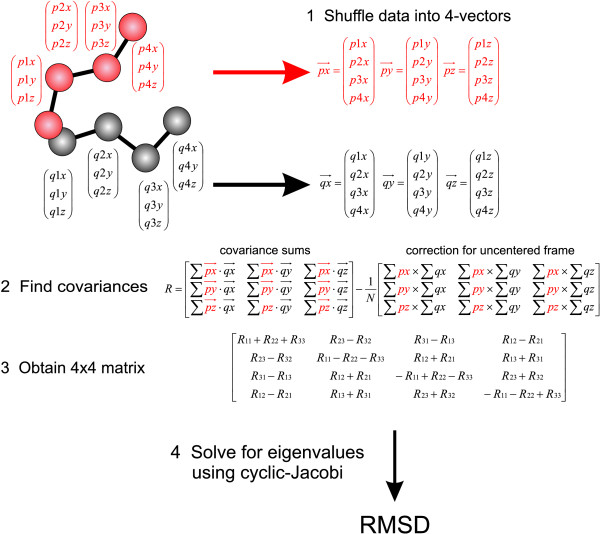
**GPU-Q-J RMSD calculation procedure**. The methodology used to calculate RMSDs on GPUs is shown. The Cartesian coordinates of the two proteins are reshuffled in 4-vectors. This allows the use of built-in dot product operations for the calculation of the covariance matrix ***R***. Because we do not center the coordinates beforehand so that their barycenters are at the origin, a second term involving the mean values of the coordinates must be subtracted. By combining the two steps, we avoid an expensive extra fetch of coordinates. Optimized 4-vector summation is used to calculate the coordinate means. The values of the covariance matrix ***R ***are maintained as double precision but the 4 × 4 matrix passed to the cyclic-Jacobi routine is single precision. This compromise increases the accuracy in some degenerate cases without sacrificing speed, as the vast majority of calculations take place as 4-vector single precision operations. The final value of RMSD obtained is identical that obtained by the CPU methods to at least 2 decimal places.

GPUs have many built-in optimizations for common graphics 4-vector operations, such as dot products and addition. The calculation of the necessary covariances was accomplished by summing the 4-vectors and dot products of 4-vectors to take advantage of these optimizations. This requires re-organizing the coordinates into suitable 4-vectors of single precision floats as shown in Figure 1. The 9 covariance matrix elements were stored as double precision variables but the 4 × 4 matrix elements were passed to the Jacobi routine as single precision. Although a completely single precision implementation is possible, this compromise increased accuracy for some degenerate cases without sacrificing speed. All methods, including GPU-Q-J, give identical values for the RMSDs to 2 decimal places. This accuracy is more than sufficient for our purposes.

The test cases for the RMSD determinations involved calculating the RMSDs for every pair of structures in an ensemble. To do this, the coordinates of all the structures are first read into a single gather stream of 4-vectors. A set of indices is provided as a regular input stream to tell the stream processor the pair of offsets of the coordinates that are to be fetched by GPU sub-process and operated upon to determine the RMSD. The resultant RMSD is written to an output stream to be sent back to the CPU. By using a slightly slower gather stream, duplicate comparisons are avoided and only the values corresponding to the upper triangle of the similarity matrix are calculated.

#### Testing and comparison of different methods

**Figure 2:**
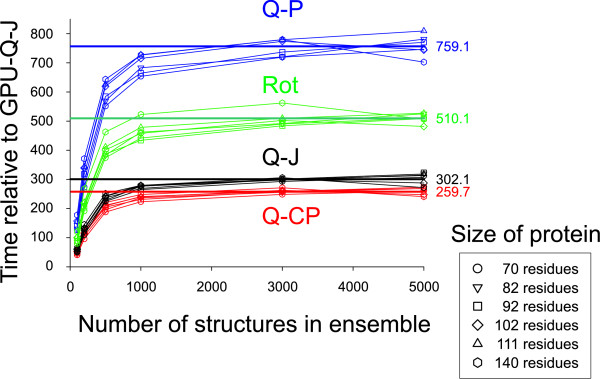
**Acceleration of RMSD calculations**. Four CPU implementations of different RMSD calculation algorithms, Quaternion Characteristic Polynomial (Q-CP), Quaternion Power (Q-P), Quaternion Jacobi (Q-J) and Rotational (Rot) were compared against our GPU implementation of the Q-J algorithm (GPU-Q-J). 6 different datasets from NRW, comprising of predictions of protein structures ranging from 70 to 140 residues in size were used as the test set. The time required to calculate the RMSDs for each pair of structures in the ensemble are displayed relative to the time required by the GPU implementation. Numbers on the right indicate the average for the 6 datasets. The results show that for ensembles of greater than 1000 structures, the overhead in setting up the GPU algorithm becomes negligible. There is a 260-fold increase in speed over the fastest CPU implementation. This increase in speed allows large ensembles of structures to be clustered quickly and relieves a major bottleneck in processing the results from NRW.

Testing was done using 6 different data sets comprising of structure predictions from NRW. The protein sequences ranged in size from 70 to 140 residues and ensemble sizes ranged from 100 to 5000. Times were recorded for the calculation of all-atom RMSDs between every pair in an ensemble of structures. For each run, the GPU times were determined by averaging 5 different timings. Timings for each of the CPU methods were then determined and expressed as a multiple of the average GPU time. The results from 6 different runs were averaged for each dataset. These results are shown in Figure 2. For all the ensemble sizes, the GPU implementation was the fastest. As the number of comparisons increases the overhead involved to use the GPU becomes negligible. For the largest ensemble size, the GPU implementation was, on average 260-fold faster than the fastest CPU method, Q-CP, and almost 800-fold faster than the slowest method, Q-P. Some of these differences are due the CPU methods not being optimized for speed. In Additional file 1: Supplemental Table S1, we also provide the actual times in seconds required to calculate the RMSDs and to read in the coordinates. Read times can be significant if structures are stored as PDB text files. However, to conserve space and bandwidth, structures from NRW are stored as binary files of torsional angle values which are then used to reconstruct the Cartesian coordinates. This entire process takes only a few seconds even for 5000 structures.

The CPU methods were compiled with gcc 4.4.1 with the optimization flags enabled. The CPU implementation of Q-J used the exact algorithm used in the GPU version, including the use of double and single precision variables. Although Q-CP was the fastest of the CPU methods by a significant margin, it was not an order of magnitude faster as previously reported [7]. The speed of the Q-CP implementation is heavily dependent on the number of iterations that is required for the Newton-Raphson method to converge. Convergence was likely slower for the divergent structures encountered in our test sets which were derived from *de novo *predictions.

## Conclusions

For most routine applications, the speed of current CPU implementations of RMSD is not an issue. As a result, with the possible exception of Q-CP, the CPU implementations are not optimized for speed and speed-ups should be obtainable with properly optimized GPU implementations of the other algorithms. Also for some GPUs the speed advantage between single precision and double precision math is not as pronounced. A stable double precision non-iterative eigenvalue solution might then be more efficient than the iterative Jacobi method that we have described. In any case, GPU-Q-J is a stable and very fast method.

Applications such as iterative density [12] and clustering [19] that require a complete pairwise similarity matrix will benefit significantly from the acceleration of the RMSD calculations. The GPU-Q-J method can be applied to more complicated structural comparison methods that use multiple RMSD determination steps [2-4]. Such methods are useful in functional annotation, where predicted structures are compared against a library of folds to help determine the function of the novel protein. Computational time is significant when these techniques are used to produce annotations on a genome scale as is being done in NRW.

Projects such as NRW create datasets so large that clustering using RMSD or other structural similarity metric is cumbersome with existing methods. With our new GPU based method, we have eliminated this bottleneck in the evaluation of the NRW datasets. In addition, we will be comparing the structures from the 60,000 sets of predictions with protein fold libraries to determine their function. Our new RMSD method can be applied to the structural comparison methods used in the annotation step as well. This will further accelerate the analysis of the 10 billions structures returned by NRW which we hope will allow us to help better understand rice genome and to potentially develop better strains of rice.

GPUs with the power of small supercomputers are becoming ubiquitous in consumer computing devices. The test system that we used is a modest one and is exceeded by many home computers used for gaming or for high definition video. These devices are being made accessible to scientific applications through community grids which link millions of volunteer nodes together. Although GPU-Q-J was developed for clustering large ensemble sets on our local servers, the method will form part of a new GPU-aware protein folding client that is in development. Such GPU-aware clients have already made an impact in projects such as Folding@home [20]. Effective GPU adaptations of routines for commonly used calculations such as the optimal superposition/RMSD are important if we are to fully utilize the enormous power being made available through the generosity of participants in projects such as NRW.

## Competing interests

The authors declare that they have no competing interests.

## Authors' contributions

LHH wrote and tested the GPU and the CPU Q-J RMSD implementations. MG assembled and maintained the NRW datasets. RS is the principal investigator for NRW and initiated the project as part of the annotation of the rice genome.

## Supplementary Material

Additional file 1 **Supplemental Table S1**. The table indicates the times in seconds, required for the different RMSD calculations for the 6 proteins from NRW ranging in size from 70 to 140 residues. In addition the times required to read in the torsional coordinates and convert them to Cartesian coordinates are indicated. Click here for file
