# Supplementary material for: GPU-Q-J, a fast method for calculating root mean square deviation (RMSD) after optimal superposition
Source: BMC Res Notes. 2011 Apr 1;4:97. doi: 10.1186/1756-0500-4-97 (PMC3087690; doi:10.1186/1756-0500-4-97)
Supplement: Additional file 1 — Supplemental Table S1. The table indicates the times in seconds, required for the different RMSD calculations for the 6 proteins from NRW ranging in size from 70 to 140 residues. In addition the times required to read in the torsional coordinates and convert them to Cartesian coordinates are indicated. [file 1756-0500-4-97-S1.DOC]

# Supplemental Materials

## Table 1

The table indicates the times in seconds, required for the different RMSD calculations for the 6 proteins from NRW ranging in size from 70 to 140 residues. In addition the times required to read in the torsional coordinates and convert them to Cartesian coordinates are indicated. Times are the average of 6 different trials, except for the GPU times which are the average of 30 trials. Standard deviations are in brackets.

Ensemble = 100 structures

Size Setup Time GPU-Q-J Q-CP Q-J Rot Q-P

70 5.1 ( 4.8) 0.01 (0.00) 0.35 (0.00) 0.43 (0.05) 0.68 (0.02) 1.07 (0.17)

82 4.32 (3.85) 0.01 (0.00) 0.43 (0.01) 0.49 (0.01) 0.81 (0.01) 1.24 (0.04)

92 4.00 (6.17) 0.01 (0.00) 0.50 (0.01) 0.56 (0.01) 0.91 (0.02) 1.44 (0.03)

103 2.12 (2.07) 0.01 (0.00) 0.57 (0.01) 0.65 (0.01) 1.07 (0.01) 1.68 (0.05)

111 1.43 (0.76) 0.01 (0.00) 0.57 (0.01) 0.65 (0.01) 1.10 (0.02) 1.67 (0.04)

140 3.09 (2.91) 0.01 (0.00) 0.71 (0.01) 0.80 (0.01) 1.49 (0.01) 2.19 (0.04)

Ensemble = 200 structures

Size Setup Time GPU-Q-J Q-CP Q-J Rot Q-P

70 1.89 (0.04) 0.01 (0.00) 1.40 (0.01) 1.61 (0.00) 2.80 (0.21) 4.00 (0.05)

82 1.81 (0.05) 0.02 (0.00) 1.73 (0.02) 1.97 (0.01) 3.45 (0.22) 4.82 (0.08)

92 1.11 (0.02) 0.02 (0.00) 2.07 (0.22) 2.35 (0.20) 3.67 (0.06) 5.7 ( 0.2)

103 1.04 (0.02) 0.02 (0.00) 2.26 (0.02) 2.60 (0.02) 4.29 (0.02) 6.6 ( 0.1)

111 1.04 (0.02) 0.02 (0.00) 2.34 (0.23) 2.60 (0.01) 4.39 (0.03) 6.8 ( 0.1)

140 1.46 (0.03) 0.02 (0.00) 2.85 (0.03) 3.31 (0.22) 6.1 ( 0.2) 8.5 ( 0.2)

Ensemble = 500 structures

Size Setup Time GPU-Q-J Q-CP Q-J Rot Q-P

70 2.21 (0.04) 0.05 (0.00) 8.7 ( 0.2) 10.4 ( 0.2) 17.3 ( 0.3) 25.5 ( 0.8)

82 2.19 (0.02) 0.05 (0.00) 10.7 ( 0.1) 12.5 ( 0.1) 20.9 ( 0.3) 31.1 ( 0.6)

92 1.57 (0.02) 0.06 (0.00) 12.4 ( 0.4) 14.3 ( 0.2) 23.4 ( 0.5) 35.3 ( 0.5)

103 1.56 (0.02) 0.07 (0.00) 14.2 ( 0.2) 16.2 ( 0.1) 26.9 ( 0.1) 41.6 ( 0.8)

111 1.62 (0.07) 0.07 (0.00) 14.2 ( 0.1) 16.5 ( 0.3) 27.9 ( 0.2) 42.5 ( 0.4)

140 2.12 (0.01) 0.08 (0.00) 18.0 ( 0.4) 20.6 ( 0.3) 37.8 ( 0.7) 52.6 ( 1.2)

Ensemble = 1000 structures

Size Setup Time GPU-Q-J Q-CP Q-J Rot Q-P

70 2.72 (0.06) 0.16 (0.02) 34.8 ( 0.4) 41.3 ( 0.5) 68.9 ( 1.1) 102.0 ( 2.9)

82 2.87 (0.04) 0.18 (0.00) 42.9 ( 0.3) 50.1 ( 0.3) 83.6 ( 0.6) 123.6 ( 4.1)

92 2.34 (0.05) 0.22 (0.03) 49.5 ( 0.5) 57.7 ( 0.3) 93.1 ( 1.6) 142.3 ( 4.6)

103 2.41 (0.03) 0.23 (0.00) 56.2 ( 0.4) 64.9 ( 0.1) 107.4 ( 1.2) 167.6 ( 5.4)

111 2.55 (0.24) 0.24 (0.00) 56.9 ( 0.5) 65.9 ( 0.3) 112.9 ( 1.6) 171.7 ( 3.7)

140 3.21 (0.03) 0.29 (0.00) 72.3 ( 1.3) 81.5 ( 0.1) 152.4 ( 2.5) 211.8 ( 2.0)

Ensemble = 3000 structures

Size Setup Time GPU-Q-J Q-CP Q-J Rot Q-P

70 5.9 ( 2.4) 1.26 (0.00) 314.1 ( 2.7) 370.3 ( 2.4) 619.4 ( 7.7) 909.1 (23.2)

82 6.5 ( 2.1) 1.52 (0.01) 388.0 ( 3.7) 451.9 ( 0.5) 746.0 ( 4.3) 1092.8 (26.1)

92 6.5 ( 2.4) 1.72 (0.01) 446.2 ( 5.2) 519.0 ( 5.3) 834.4 (15.9) 1270.9 (22.2)

103 6.4 ( 0.8) 1.95 (0.02) 512.1 (15.7) 585.0 ( 2.6) 980.2 (13.2) 1510.2 (43.6)

111 6.4 ( 0.8) 1.97 (0.07) 511.1 ( 3.0) 591.1 ( 2.2) 1005.6 (12.1) 1537.0 (17.6)

140 8.0 ( 0.7) 2.40 (0.01) 649.8 (13.5) 733.0 ( 4.9) 1346.8 (12.7) 1867.9 (25.7)

Ensemble = 5000 structures

Size Setup Time GPU-Q-J Q-CP Q-J Rot Q-P

70 9.8 ( 3.7) 3.37 (0.04) 875.8 ( 4.8) 1035.5 ( 6.6) 1723.3 ( 5.8) 2517.7 (62.4)

82 10.8 ( 2.6) 3.98 (0.01) 1070.4 ( 5.1) 1253.7 ( 5.8) 2092.4 (23.0) 3112.8 (55.3)

92 13.0 ( 5.0) 4.54 (0.08) 1239.1 (14.1) 1445.3 (23.5) 2308.5 (15.4) 3496.3 (33.8)

103 10.9 ( 1.6) 5.78 (1.24) 1417.0 (10.3) 1647.0 (15.7) 2756.8 (42.2) 4256.0 (128.)

111 12.9 ( 5.2) 5.30 (0.62) 1420.9 (16.4) 1656.5 (17.9) 2788.4 (23.0) 4280.7 (87.3)

140 14.9 ( 2.6) 7.56 (2.13) 1813.8 (17.2) 2047.0 ( 7.0) 3817.8 (50.6) 5292.1 (92.5)
